# Etching-Induced Surface Reconstruction of NiMoO_4_ for Oxygen Evolution Reaction

**DOI:** 10.1007/s40820-022-01011-3

**Published:** 2023-01-09

**Authors:** Jinli Zhu, Jinmei Qian, Xuebing Peng, Baori Xia, Daqiang Gao

**Affiliations:** Key Laboratory for Magnetism and Magnetic Materials of MOE, Key Laboratory of Special Function Materials and Structure Design of MOE, Lanzhou University, Lanzhou, 730000 People’s Republic of China

**Keywords:** Etching, Surface reconstruction, Cation deficiencies, OER

## Abstract

Rational reconstruction of oxygen evolution reaction (OER) pre-catalysts and performance index of OER catalysts are crucial but still challenging for universal water electrolysis. Herein, we develop a double-cation etching strategy to tailor the electronic structure of NiMoO_4_, where the prepared NiMoO_4_ nanorods etched by H_2_O_2_ reconstruct their surface with abundant cation deficiencies and lattice distortion. Calculation results reveal that the double cation deficiencies can make the upshift of *d*-band center for Ni atoms and the active sites with better oxygen adsorption capacity. As a result, the optimized sample (NMO-30M) possesses an overpotential of 260 mV at 10 mA cm^−2^ and excellent long-term durability of 162 h. Importantly, in situ Raman test reveals the rapid formation of high-oxidation-state transition metal hydroxide species, which can further help to improve the catalytic activity of NiMoO_4_ in OER. This work highlights the influence of surface remodification and shed some light on activating catalysts.

**Supplementary Information:**

The online version contains supplementary material available at 10.1007/s40820-022-01011-3.

## Highlights

- Double-cation etching induces abundant vacancies serving as active sites and accelerates the surface reconstruction.
- NMO-30M with cation deficiencies and oxygen vacancies exhibits outstanding OER performance and remarkable stability.
- *In situ* Raman spectroscopy directly captures the surface reconstruction process.

**Supplementary Information:**

The online version contains supplementary material available at 10.1007/s40820-022-01011-3.

## Graphical abstract

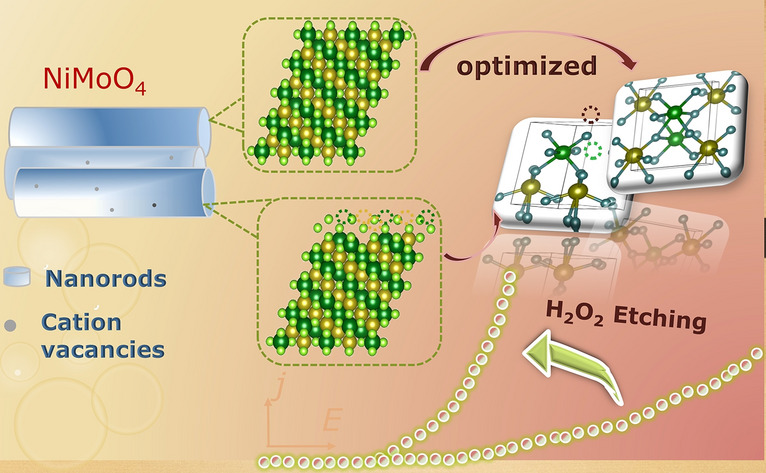

## Introduction

Sustainable energy strategies are expected not only to meet the growing demand for energy but also to alleviate the greenhouse gas emissions increasing [1–3]. Water electrolysis technologies can achieve zero CO_2_ emission and yield massive hydrogen with high purity (> 99.9%), and thus underlying serves as an imperative part in future renewable energy conversion and storage systems [4–7]. Electrocatalytic water splitting, which produces hydrogen from H_2_O without extra costs, has emerged as a representative strategy for intermittency, especially with the potentiality of urging the process by electric power generated from sustainable sources [8–10]. However, the sluggish anodic oxygen evolution reaction (OER) is the primary bottleneck in electrochemical water splitting, due to its large overpotential and slow reaction dynamics relating multiple electron and proton transports [11–14]. Therefore, enhancing the catalytic activity and expediting the dynamics characteristic on anode are eagerly desired for realizing high-performance electrocatalysts.

Although a few noble metal catalysts (e.g., IrO_2_ and RuO_2_) exhibit excellent electrocatalytic capacity for OER reactions, it is still challenging for undertaking large-scale applications owing to their insufficient reserves and poor stability [15–17]. Up to now, multitudinous noble metal and non-noble metal materials (e.g., transition metals and their derivatives) have gained substantial attention and been produced as high-efficiency catalysts for oxygen evolution reactions [18–25]. Especially, for Ni-based materials, electro-induced oxidation on their surfaces generally triggers reconstruction to form hydroxides because of their unique electronic structures for binding intermediates, which have been identified as real active species [26]. Recently, Liu et al. [27] reported that the deeply reconstructed catalyst (NiOOH) with sufficient active species had achieved high-mass-activity catalysis. Mai et al. also uncovered that the thermally induced complete reconstruction of molybdate pre-catalysts at 51.9 °C showed excellent OER performance [28]. Zhao et al. revealed that a vertically interlaced NiFeP/MXene catalyst could tune the electronic structure and expose more active sites in the catalytic system [29]. Noting that these post-OER catalysts generally show a near-surface reconstruction structure within thin layer and exhibit incompletely developed catalytic activity owing to their limited near-surface reaction regions [12, 30]. Thus, there exist challenges in extending the reconstruction layer and understanding the mechanisms of dynamic reconstruction. Recently, impressive performance has been observed in pre-catalysts with self-reconstruction, which is induced by an etching effect of the ions, endowing the derived catalysts with a large amount of active species [31, 32]. As previously reported, the ion leaching behaviors in pristine materials could trigger favorable OH^−^ adsorption on catalyst surface and thus accelerate OER processes [33–35]. Han et al. [36] unveiled the LiNiO_2_ (LNO) model containing a series of controllable cation-vacancy exhibits an extremely low overpotential at 10 mA cm^−2^ along with the change of geometric structure in catalyst. Xiao et al. [37] reported an in situ electrochemical cation-exchange method to create the cation deficiencies in MnO_2_. Thus, pristine materials will sacrifice the part of surface crystalline by the loss of ions, to form amorphous low-conductivity regions and lead to the collapse of materials, accelerating the electrolyte infiltration [33, 34, 38].

Inspired by above statement, here we report a novel surface-electronic-structure reconstructing strategy in NiMoO_4_ via efficient double-cation etching, where Mo^6+^ with high electronegativity has been proved to effectively improve the Ni oxidation state [39]. Experimental characterizations reveal rich cation deficiencies and lattice distortion on account of the metal dissolving after H_2_O_2_ etching. The optimized sample (NMO-30M) exhibits an excellent overpotential of 260 mV at 10 mA cm^−2^ and a Tafel slope of 85.7 mV dec^−1^. Density functional theory (DFT) calculation results indicate that the *d*-band center moves closer to Fermi level in the case of existing surface double cation deficiencies, which accelerates the kinetics rate and catalytic activity. At the same time, the model with surface double cation deficiencies (NMO-V_NiMo_) exhibits a lower calculated overpotential of *η* = 0.84 V for the rate-determining step than that of pristine NiMoO_4_ (NMO) with *η* = 1.15 V, clearly indicating that NMO-V_NiMo_ displays the better OER activity. The study offers a new insight to further understand the electrocatalytic mechanism of surface reconstruction.

## Experimental Section

### Synthesis of NMO, NMO-30M and NMO-50M

Typically, we synthesized NiMoO_4_ by a simple hydrothermal process [40]. The precursors were collected and calcinated at 450 °C for 2 h under Ar atmosphere (90 sccm) with a heating rate of 5 °C min^−1^. Thus, the pristine NiMoO_4_ was obtained and named as NMO. Then, 5 μL H_2_O_2_ was dropped into 2.47 mmol L^−1^ NiMoO_4_ aqueous solution and maintained for 30 and 50 min continuously for etching. After that, two etched samples were obtained and named as NMO-30M and NMO-50M, respectively.

### Material Characterizations

Transmission electron microscope (TEM, Tecnai TM G2 F30, FEI, USA) was employed to reveal the surface reconstruction of the as-synthesized catalysts. The surface morphology and microstructure of catalysts was probed by scanning electron microscopy (SEM, Thermo Scientific, Apreo S). X-ray diffraction (XRD, X’ Pert PRO PHILIPS with Cu Kα radiation) and Raman spectrometer (Lab-RAM HR Evolution) were carried out to decide the phase structure. The cation amounts of the samples were detected by an inductively coupled plasma optical emission spectrometer (ICP-OES, Agilent 5110, USA). The elemental composition and the valence state of the catalysts were studied by X-ray photoelectron spectroscopy (XPS, Kratos Axis Ultra). Elemental spectra and mappings were obtained by an energy-dispersive spectrum analyzer (EDS, Bruker SuperX). Temperature programmed desorption of oxygen gas (O_2_-TPD) was measured with a Micromeritics AutoChem II 2920 chemisorption analyzer. In addition, microscopy images, selected area electronic diffraction (SAED) patterns, elemental mapping, and linear scanning analysis were explored with Titan Gubed Themis G2300 scanning/transmission electron microscopes.

### Calculation Details

DFT calculations were computed by Vienna ab initio simulation package (VASP) with the Projected Augmented Wave (PAW) method [41, 42]. The exchange correlation energy was described by generalized gradient approximation (GGA) method with Perdew–Burke–Ernzerhof (PBE) function [43]. The energy cutoff for the plane-wave basis was set with 400 eV, and the convergence was 1 × 10^−6^ eV, respectively. The force component was 0.01 eV Å^−1^. Brillouin zone was selected using 10 × 10 × 11 k-point mesh generated on the basis of Monkhorst–Pack scheme. The calculation details of the OER four-step reaction are shown in the supplementary materials.

### Electrochemical Measurements

The electrochemical measurement of the catalysts was conducted on an electrochemical workstation (CHI770e) using a standard three-electrode system in 1 M KOH electrolyte. The Ag/AgCl electrode and a platinum foil filled with 1 M KOH electrolyte are applied as the reference electrode and counter electrode, respectively. For the working electrode, powder of sample (3 mg) and carbon black (3 mg), Nafion (5 wt%, 30 μL) and DMF (1470 μL) were mixed and sonicated for 5 h to form homogeneous electrochemical test solution with good dispersion. Linear sweep voltammetry (LSV) curves were measured in 1 M KOH electrolyte at a sweep rate of 2 mV s^−1^. The potentials tested by CHI770e were referenced to reversible hydrogen electrode (RHE) scale according to the Nernst equation of *E*_RHE_ = *E*_Ag/AgCl_ + 0.197 + 0.059 × pH and *iR*-correctioned. The electrolyte resistance *R*_s_ and charge-transfer resistance (*R*_ct_) were determined by specific electrochemical impedance measurement (EIS) for different catalysts from 10 kHz to 0.1 Hz. The double-layer capacitances (*C*_dl_) were obtained from the cyclic voltammetry (CV) curves received at a voltage of 0–0.2 V versus RHE at scan rates of 20, 40, 60, 80, 100 and 120 mV s^−1^.

## Results and Discussion

### Material Synthesis and Characterization

**Fig. 1:**
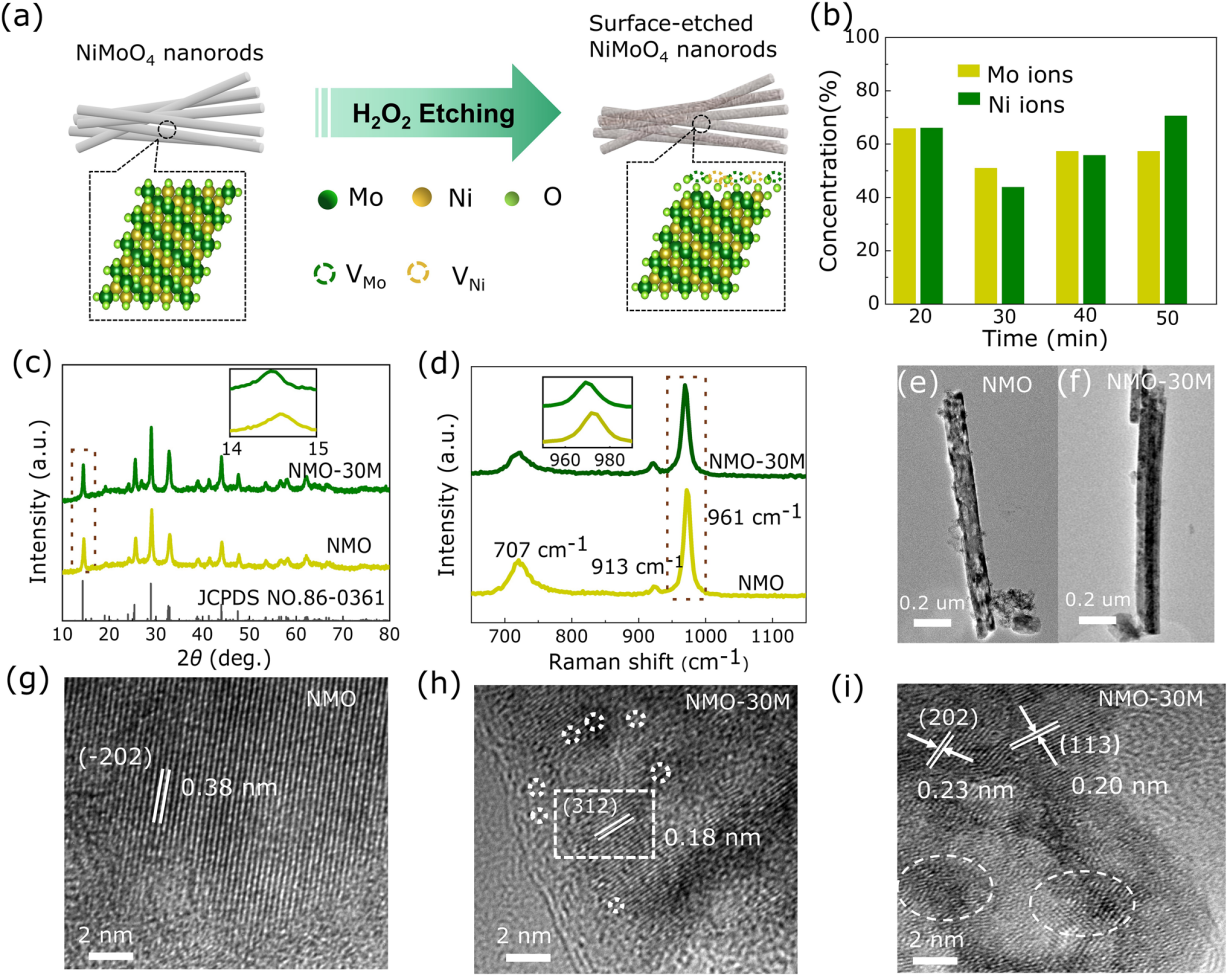
Structure, morphology and phase composition of catalysts. **a** Diagram of synthesis process for NMO-30M and NMO-50M. **b** Contents of Ni and Mo in the supernatant after etching of NiMoO_4_ (plotted from ICP results). **c** XRD pattern and **d** Raman spectra of as-prepared NMO and NMO-30M. The insets are the zoomed images of for XRD and Raman, respectively. The TEM images of **e** NMO and **f** NMO-30M. High-resolution TEM images of **g** NMO and **h**, **i** NMO-30M

Pristine scheelite-type NMO, NMO-30M and NMO-50M are synthesized by hydrothermal method, and the etching schematic diagram is shown in Fig. 1a. The atomic ratio is obtained by ICP test, during which the supernatant containing both Ni and Mo cations after etching in different times are detected (Fig. 1b). It should be discovered that generated two types of cations (Ni, Mo) deficiencies in NiMoO_4_ after H_2_O_2_ etching (Table S1). Figures 1c and S1 display the powder XRD patterns of the products. Both NMO and NMO-30M possess good crystallinity, consistent with the standard scheelite phase (JCPDS No. 86-0361) with space group C2/m. Moreover, the XRD Rietveld refinement [44] method is applied to verify the crystal structure of NMO and NMO-30M in Fig. S2. The fitting parameters in Table S2 and calculated patterns indicate acceptable correlation between observed and calculated XRD patterns. Exceptionally, the lattice constant and volume increase slightly owing to the cation deficiencies and lattice structure variation after etching, which is consistent with the result of the inset in Fig. 1c. It reveals that the diffraction peaks of (100) plane in NMO-30M shift to the lower degree (from 14.6° to 14.3°). Besides, Raman spectra shown in Figs. 1d and S3 manifest the peaks of NMO and NMO-30M locating at 961 and 707 cm^−1^, which are ascribed to the symmetric stretching of Mo–O and Ni–O–Mo bonds of NiMoO_4_ [45, 46], respectively. The peak at 913 cm^−1^ is assigned to the asymmetric stretching of Mo–O bonds [47] and there is also going to be at a lower wavenumber in inset from 972.1 to 969.3 cm^−1^, consistent with XRD results. Subsequently, the morphology and the lattice fringes of samples are characterized by SEM, TEM and high-resolution TEM (HRTEM). As shown in Figs. S4, S5 and 1e, f, the samples exhibit the typical nanorods morphology and the surface of NMO is smooth with an average diameter of ~ 200 nm. HRTEM images in Figs. 1g and S6 reveal the lattice fringes of 0.38 and 0.40 nm, which match well with the (− 202) and (111) planes of NMO, respectively. Contrastively, the surface region of the NMO-30M becomes loose and generates deficiencies on account of the metal exsolution during the etching process, illustrating the existence of cation (Ni and Mo) deficiencies (Fig. 1f, h). In addition, there are some lattice distortions at the interfaces due to the structure termination, as shown in Figs. 1i and S7. The element distribution in NMO, NMO-30M and NMO-50M is displayed by EDX elemental mapping (Fig. S8), wherein all the elements are distributed evenly.

### Electronic Structure Analysis

**Fig. 2:**
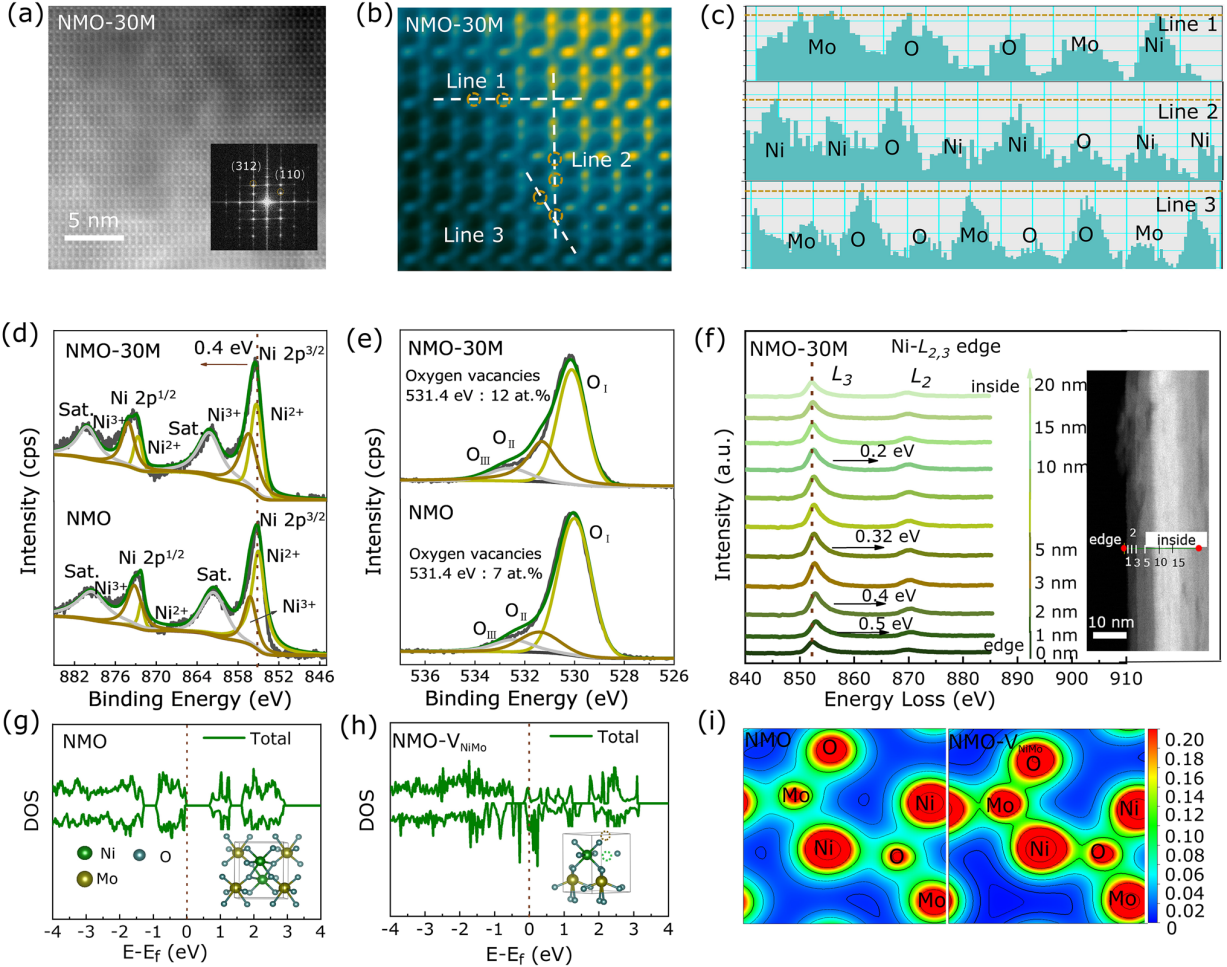
Electronic structure analysis based on experimental results and theoretical calculation. **a** Original and **b** colored HAADF-STEM image and of NMO-30M. The fast Fourier transform (FFT)-filtered atomic resolution image is shown in the inset of **a**. **c** Corresponding intensity profiles of line section. High-resolution XPS spectra of **d** Ni 2*p* and **e** O 1*s* for NMO and NMO-30M. **f** Ni *L*-edge EELS spectra among different regions (STEM image) throughout etching. DOS of **g** pristine NMO and **h** NMO-V_NiMo_. **i** The charge density diagram of NMO and NMO-V_NiMo_

Furthermore, we utilize the high-angle annular dark-field scanning transmission electron microscopy (HAADF-STEM) measurement to explore the atomic structure of NMO-30M. Figure 2a shows the high-resolution atomic patterns, and bright dots in an orderly arrangement can be observed, consistent with the lattice planes. Also, the clear diffraction spots in SAED pattern, shown in the inset of Fig. 2a, demonstrate the crystalline of NMO-30M. To further verify the lattice structure of NMO-30M, HAADF-STEM imaging method is conducted. Figures S9 and 2b present original and colored HAADF-STEM images for NMO-30M, respectively, in which a local area of atomic lattice is magnified. Depending on the difference of brightness, three types of atoms could be observed, which involves their atomic number [48]. The brightest dots in Fig. 2b can be recognized to be Mo atoms, and the slightly darker ones are Ni atoms. Noting that some dullish dots scattered in the corners between Mo and Ni atoms are O atoms. Simultaneously, all the atomic spots are well arranged. Figure 2c displays three-line profiles in Fig. 2b with disparate orientation, where the slightly weaker atomic intensity can be identified, which are ascribed to the cations (Ni and Mo) and oxygen defects in the NMO-30M lattice. To understand the oxidation states of atom in catalysts induced by etching, XPS method is undertaken. As for the Ni 2*p* spectrum of NMO, peaks at 872.4 and 873.1 eV, 855.3 and 856.6 eV are identified to the Ni 2*p*_1/2_ and Ni 2*p*_3/2_ doublets, respectively (Fig. 2d), accompanied with two satellite peaks at 879.3 and 860.4 eV [49]. The peak positions of Ni 2*p* for NMO-30M shift to the higher binding energy by 0.4 and 0.2 eV for NMO-50M (Fig. S10a), demonstrating that the valence state of Ni rises and more Ni^3+^ exists in NMO-30M and NMO-50M [50]. The ratio of Ni^3+^/Ni^2+^ for NMO-30M is 0.65, which is higher than that of NMO and NMO-50M in Table S3. As shown in Fig. S10b of Mo 3*d* XPS spectrum, the optimal deconvolution of the Mo 3*d* profile displays two sets of doublets which could be described to Mo^6+^ and Mo^5+^ [51, 52]. The intensity ratio of Mo^5+^ in NMO-30M exhibits remarkable enhancement, corresponding to the augment of the etched cation (Ni and Mo) and manifesting the greatly increasing content of oxygen defects during etching process [53]. The O 1*s* spectra for three samples (Figs. 2e, S10c) can be fitted to metal–oxygen (O_I_) at 529.7 eV, and oxygen vacancy (O_II_) as well as surface adsorbed oxygen (O_III_) exist at 531.4 and 532.6 eV, relatively [53–55]. As for NMO-30M, the concentration of oxygen vacancies determined by XPS is 12 at.%, which is much larger than in the NMO (7 at.%) and NMO-50M (9 at.%). Magnetic properties of NMO and NMO-30M samples are observed at room temperature by VSM measurements, and the consequent magnetic hysteresis loops of them are shown in Fig. S11. NMO-30M shows the ferromagnetic behavior, while NMO reveals paramagnetic, implying the changes of spin state for Ni ions in NMO-30M owing to appearance of Ni^3+^ [56, 57].

According to the electron energy loss spectroscopy (EELS) results shown in Fig. 2f, when starting line scanning from the edge to the inside gradually, the Ni *L*_3_/*L*_2_-edge will change significantly. As the line scanning depth is 1–2 nm, there is a remarkably position shift of the Ni *L*_3_/*L*_2_-edge with 0.5 and 0.4 eV, indicating that the electronic structure of Ni has changed after etching. With the depth increasing, the Ni *L*_3_/*L*_2_-edge shifting to high binding energy decreases and eventually disappears at 15 nm. Also, the Bader charge analysis is shown on Ni cation, and the evaluated average valence charge of Ni cation reduces from 8.63 eV (in NMO) to 8.40 eV (in NMO-V_NiMo_) (Table S4), corresponding to the result of XPS. Note that the electronic state of Mo is not affected in EELS line scanning (see Fig. S12). Figures S13 and S14 exhibit the HAADF images and elemental mappings of Ni, Mo and O as well as the contents of each element within the selected area.

To further probe the effects of etching on the catalytic performance, the DFT calculations are performed to investigate the cases: pristine NMO, and NMO with Ni/Mo deficiencies (NMO-V_NiMo_). As shown in Fig. 2g, h, the density of states (DOS) results indicate that the absence of occupied electronic state near the Fermi level in NMO reveals the poor electronic conductivity of it, while the introduction of Ni and Mo deficiencies can boost the conductivity and make discrete energy levels becoming continuous in NMO-V_NiMo_, and the calculated gap is reduced from 0.67 eV (NMO) to 0 eV (NMO-V_NiMo_). Furthermore, there is a larger DOS intensity at the Fermi level for NMO-V_Mo_ (Mo deficiencies) and NMO-V_Ni_ (Ni deficiencies) in Fig. S15, from which the gaps are evaluated to be 0.21 and 0.05 eV, respectively. Figure 2i displays the charge-density distribution of NMO and NMO-V_NiMo_. We can see that the charge distribution around Mo and Ni atoms is stronger in NMO-V_NiMo_, suggesting the better electronic conductivity of it.

### Electrochemical OER Performance

**Fig. 3:**
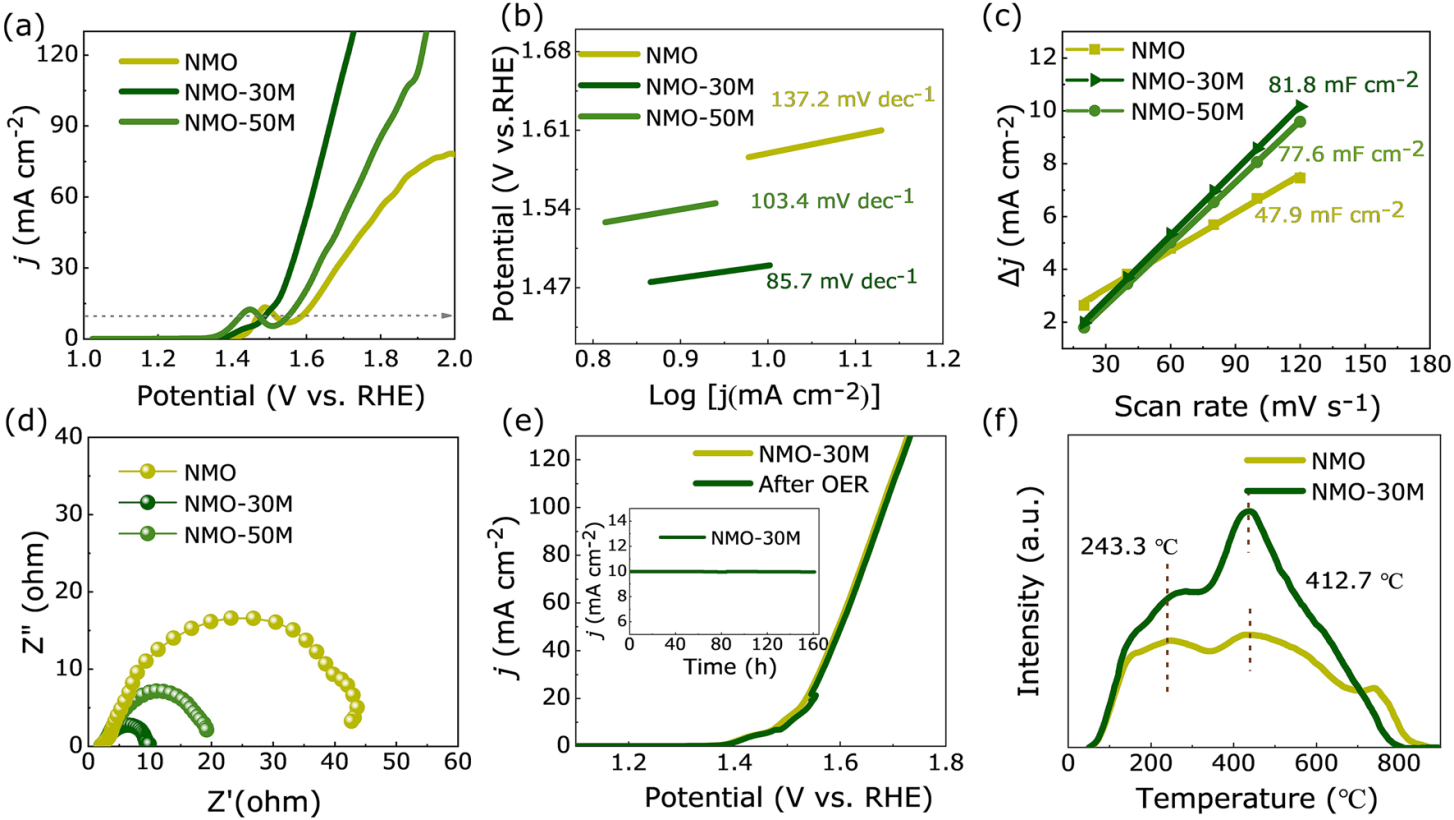
Electrochemical performance tests of catalysts. **a** Polarization curves and **b** the corresponding Tafel plots of NMO, NMO-30M and NMO-50M. **c** *C*_dl_ obtained by CV curves at 0.1 V versus Ag/AgCl. **d** EIS spectra of NMO, NMO-30M and NMO-50M. **e** LSV curve of NMO-30M after cycles and the inset is *i*–*t* curve of NMO-30M at a potential of 1.6 V versus RHE. **f** O_2_-TPD profiles of NMO and NMO-30M

The OER performances of catalysts are measured by a three-electrode system in 1 M KOH electrolyte. The polarization curves obtained from LSV with a scan rate of 2 mV s^−1^ of the three samples, herein all the potential values presented in this work are *iR*-corrected and referenced to the RHE. The polarization curves are shown in Fig. 3a. It can be seen that an oxidation of Ni^2+^ to Ni^3+^ occurs in the potential range of 1.35–1.55 V and the NMO shows a high overpotential of 360 mV at 10 mA cm^−2^, while the values of overpotential are reduced to 260 and 323 mV in NMO-30M and NMO-50M, respectively. It illustrates that the catalytic properties are facilitated after etching. Besides, as shown in Fig. 3b, the Tafel slopes obtained from LSV curves are plotted and estimated to reflect their OER dynamics. The Tafel slope of NMO-30M is about 85.7 mV dec^−1^, much smaller than that of NMO nanorods (137.2 mV dec^−1^) and NMO-50M (103.4 mV dec^−1^), indicating the most favorable OER dynamics in NMO-30M. The *C*_dl_ is estimated and acquired from CV curves (Fig. S16) because it is positively related with the electrochemically active surface areas (ECSA) of catalysts [58]. The *C*_dl_ values of NMO, NMO-30M and NMO-50M are fitted to be 47.9, 81.8 and 77.6 mF cm^−2^, respectively. And the effective ECSA of NMO-30M is about twice as larger as that of NMO. The results suggest that metal-ions etching and co-leaching roughen the surface of the nanorods, exposing more active sites and the surface reconstruction ensues during OER process. The *R*_ct_ obtained from the EIS is shown in Fig. 3d. NMO-30M shows a smaller semicircle radius than NMO and NMO-50M, accompanied with a smaller value of *R*_ct_ (Fig. S17), suggesting that it possesses the fastest reaction kinetic. The stability of catalyst is of great significance for its practical application. The cycling testing of NMO-30M in OER is also carried out to evaluate the surface structure stability. As shown in Fig. 3e, the overpotential at 10 mA cm^−1^ just changes about 5 mV after cycles and the time-varying current density curve of NMO-30M (inset) remains unchanged at 1.6 V versus RHE for 162 h, illuminating the catalyst represents prominent stability during electrochemical process. In addition, the XRD patterns (Fig. S18) of NMO-30M before and after stability test for 162 h display that the phase structure does not change apparently. The NMO-30M still maintains the nanorod structure with robust subunits and element compositions (Fig. S19), further suggesting that the NMO-30M electrode possesses excellent long-term stability. Nevertheless, the brightness of Mo is dimmed. Moreover, as for the XPS results of three samples before and after OER shown in Fig. S20, the peak positions of Ni 2*p* shift to the higher binding energy about 0.2 eV after OER, demonstrating that the valence state of Ni increases, which is beneficial for the formation of NiOOH in OER process. The intensity of Mo after OER becomes weaker than before due to the surface reconstruction.

Furthermore, O_2_-TPD is carried out to estimate the oxygen adsorption ability of the catalysts. As shown in Fig. 3f, it can be seen that two peaks centered at the temperatures of 243.3 and 412.7 °C for NMO and NMO-30M, respectively, which is attributed to the weakly adsorbed oxygen species [59]. NMO-30M shows high-intensity peaks, reflecting effectively adsorb/desorb oxygen ability. In electrocatalysis, the wettability of catalysts is closely relevant to the interaction between the catalysts and electrolytes [60]. The results in Fig. S21 display that the contact angle of NMO-30M is 26.1°, smaller than those in NMO-50M (35.2°) and NMO (54.4°), revealing that cation deficiencies enrich the contact of reactants. Besides, the ratio of H_2_ and O_2_ collected by drainage method is about 2:1, and the Faraday efficiency is nearly 97% shown in Fig. S22.

**Fig. 4:**
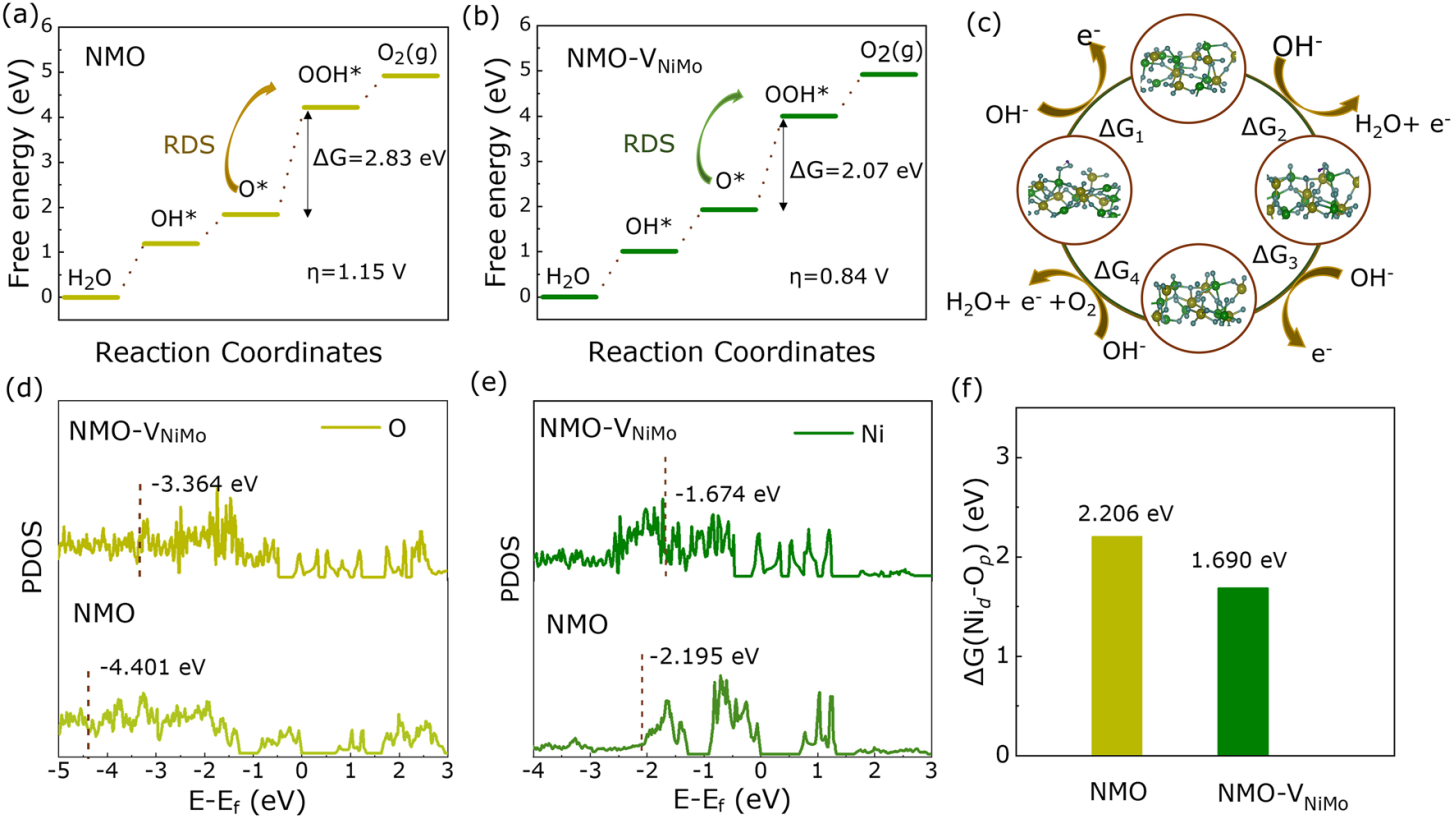
DFT calculation of NMO and NMO-V_NiMo_. Free energy diagram of OER steps on **a** NMO and **b** NMO-V_NiMo_. **c** Schematic of a four-step mechanism of OER. Partial DOS of **d** NMO and **e** NMO-V_NiMo_. **f** Energy difference of Ni *d* and O *p* in NMO and NMO-V_NiMo_

The total OER progress could be generalized in four elementary reaction steps consisting of three pivotal intermediates: *OH, *O, and *OOH [61]. Figure 4c exhibits the optimized geometric model of diverse intermediates correlated to the reaction pathway, and corresponding energy profiles are depicted in Fig. 4a, b. The conversion of *OOH to *O intermediates is the rate-determining step (RDS) for NMO-V_NiMo_, with the lower energy barrier of 2.07 eV than NMO (2.83 eV). The overpotential (*η*) calculated by DFT calculations is 0.84 V for *O + H_2_O (l) ⇋ *OOH + H^+^  + e^−^, which is smaller than that of pristine NMO (*η* = 1.15 V) [6]. Consequently, we conclude that the decrease in the RDS in favor of the improvement of OER performances. In addition, as shown in Fig. 4d, e, the *d*-band centers of Ni *d* and O *p* in NMO-V_NiMo_ are closer to Fermi level than NMO, correlated with strong adsorption for oxygen intermediates. As shown in Fig. 3f, the energy difference between the Ni *d* and O *p* band center decreases to 1.690 eV after introducing of double cation deficiencies, signifying that the Ni 3*d-*O 2*p* covalency in NMO-30M has been promoted, which leads to the lower potential of the four steps in OER process [62]. The *d*-band center and the energy difference of NMO-V_Ni_ and NMO-V_Mo_ are exhibited in Figs. S23, S24. It shows that the results of NMO-V_Ni_ and NMO-V_Mo_ are inferior to NMO-V_NiMo_.

### Identification of Surface Reconstruction

**Fig. 5:**
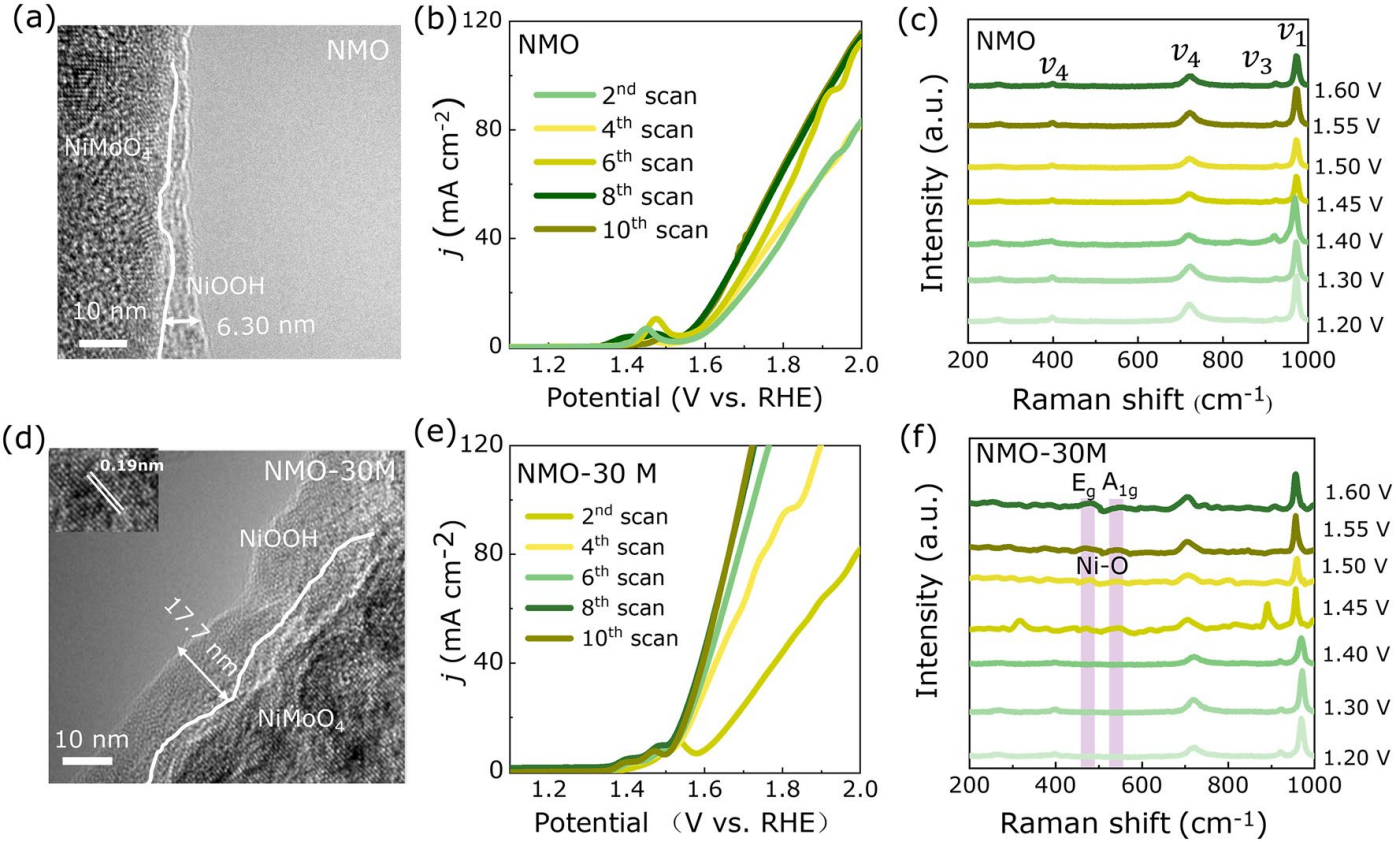
Self-reconstruction of NMO and NMO-30M in 1 M KOH during OER. The morphology structures of **a** NMO and **d** NMO-30M after self-reconstruction. The self-reconstruction process of **b** NMO and **e** NMO-30M. *In situ* Raman spectra of **c** NMO and **f** NMO-30M tested in 1 M KOH at different potentials versus RHE during OER process

To survey the dynamic surface reconstruction process under OER reaction conditions, the TEM characterizations after OER and LSV with different scans of NMO and NMO-30M are carried out. As shown in Fig. 5a, the reconstruction layer of ~ 6.3 nm is observed in NMO. The reconstruction degree is deepened for NMO-30M, up to ~ 17.7 nm owing to the etching and co-leaching of cation (Fig. 5d), which causes a loose reconstruction layer and accelerates its self-reconstruction. The LSV pattern of NMO-30M changes faster than NMO and possesses lower overpotential during OER process (Fig. 5b, e). As well known, the reconstruction reaction takes place in and involves an alkaline solution, and electrochemical tests enable the accelerated surface reconstruction and enrichment of NiOOH intermediates during electrooxidation [63]. Here, Mo and Ni species are leached in etching process, giving rise to the loose surface regions in the nanorods, which is beneficial for electrolyte penetration and the formation of NiOOH.

In order to investigate the reconstruction mechanism of NMO and NMO-30M, an in situ electrochemistry-Raman coupling system is applied. As shown in Fig. 5c, firstly, we explore the NMO sample under bias voltages. When bias voltage gradually increases on NMO, all of detectable bands are ascribe to Mo–O vibrations with 385–705, 815–910 and 960 cm^−1^ assigned to ${v}_{4}$, ${v}_{3}$, and ${v}_{1}$(2A_g_) vibration modes of Mo–O in NMO, respectively [64]. However, NMO-30M in Fig. 5f appears two faint bands at 473 and 553 cm^−1^, which is attributed to the *E*_g_ (Ni^3+^–O) bending vibration mode and *A*_1g_ (Ni^3+^–O) stretching vibration mode, indicating the formation of NiOOH [65, 66]. Combined with the results of XPS, we realize the oxidation state of Ni rises and more Ni^3+^ exists in NMO-30M after etching, and deduce that the increase in Ni enhances the formation of NiOOH intermediates in alkaline solution [27]. As for NMO, the absent Raman peaks of NiOOH are ascribed to its thin layer of the surface [67]. Besides, when bias voltages are added and gradually increase on NMO-30M, the intensity of Mo–O bands (385 and 910 cm^−1^) accordingly fades away and completely disappears at 1.65 V versus RHE owing to the dissolution of MoO_4_^2−^ during the reconstruction process under OER conditions, which originates from dissolution of Mo species [68]. The results are consistent with the consequence of EDX mappings and XPS results after OER. According to the above analysis, it can be summarized that etching distorts the surface structure of NMO-30M nanorods and undergoes the self-reconstruction during OER process, including the rapid dissolution of MoO_4_^2−^ and the fast formation of NiOOH simultaneously, thus boosting overall OER activity.

## Conclusion

In summary, we have successfully improved the electrocatalytic performance of the NiMoO_4_ catalyst by etching strategy. Theoretical results demonstrate that the existences of double cation deficiencies are beneficial for charge transfer during the OER process because of the narrowed band and changed *d*-band center closer to the Fermi level. Experimental results reveal that the dynamic of NMO-30M has been greatly improved and the charge transfer impedance is reduced by half. Surface cations deficiencies and large areas of lattice mismatch are conducive to the infiltration of the electrolyte and form the reconstructed layer of NiOOH during the OER process, and the in situ Raman technique well demonstrates the reconstruction process. This work demonstrates that surface reconstruction is one of the desirable methods to improve the performance of oxides catalysts.

### Supplementary Information

Below is the link to the electronic supplementary material.

Supplementary file1 (PDF 1659 KB)
