# Supplementary material for: Etching-Induced Surface Reconstruction of NiMoO4 for Oxygen Evolution Reaction
Source: Nanomicro Lett. 2023 Jan 9;15:30. doi: 10.1007/s40820-022-01011-3 (PMC9829944; doi:10.1007/s40820-022-01011-3)
Supplement: Supplementary file 1 — Supplementary file1 (PDF 1659 KB) [file 40820_2022_1011_MOESM1_ESM.pdf]

Supporting Information for

Etching Induced Surface Reconstruction of $NiMoO_4$ for Oxygen Evolution Reaction

Jinli Zhu[1], Jinmei Qian[1], Xuebing Peng[1], Baori Xia[1,] * and Daqiang Gao[1,] *

[1]Key Laboratory for Magnetism and Magnetic Materials of MOE, Key Laboratory of Special Function Materials and Structure Design of MOE, Lanzhou University, Lanzhou 730000, People's Republic of China

*Corresponding authors. E-mail: xiabr@lzu.edu.cn (Baorui Xia); gaodq@lzu.edu.cn (Daqiang Gao)

S1 Computational Methods

The micro kinetic process of water-splitting reaction is modeled with the approach used by Norskov and coworkers [S1-S3]. Two half-reaction equations, i.e., OER and HER, are listed as below:

$$2H_2O \rightarrow O_2 + 4H^+ + 4e^- \quad (S1)$$

$$4H+ + 4e^- \rightarrow H_2 \quad (S2)$$

For OER, it is decomposed into four one-electron steps OER-1 to OER-4 with each step generates one H^+ and an electron, listed as below:

$$* + H_2O(l) \rightarrow OH* + (H^+ + e^-) \quad (S3)$$

$$OH* \rightarrow O* + (H^+ + e^-) \quad (S4)$$

$$O* + H_2O(l) \rightarrow OOH* + (H^+ + e^-) \quad (S5)$$

$$OOH* \rightarrow * + O_2(g) + (H^+ + e^-) \quad (S6)$$

The Gibbs free energy change (ΔG) for each elemental step is defined as [S4,S5]:

$$\Delta G = \Delta E + \Delta ZPE - T\Delta S + \Delta G_U + \Delta G_{\mathrm{pH}} \quad (S7)$$

where ΔE and ΔZPE are the adsorption energy based on density functional theory calculations and the zero-point energy correction, respectively. T, ΔS, U, and ΔG_{pH} represent the temperature, the entropy change, the applied electrode potential, and the free energy correction of the pH, respectively.

As a generally-accepted OER activity descriptor, the overpotential (η) for a chemical reaction can be calculated as follow:

$$\eta = \max[\Delta G_1, \Delta G_2, \Delta G_3, \Delta G_4]/e - 1.23[v] \quad (S8)$$

S2 Supplementary Figures

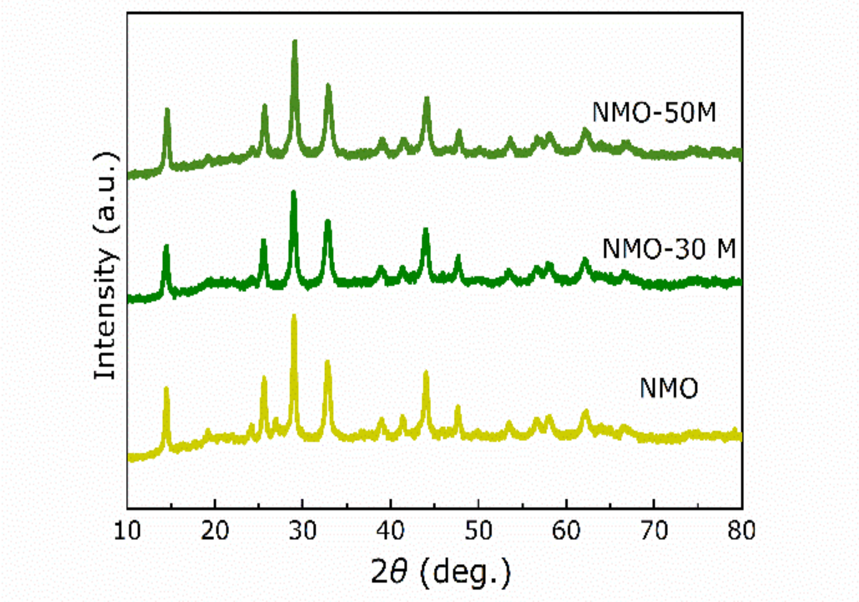

Fig. S1 XRD pattern for NMO, NMO-30M and NMO-50M

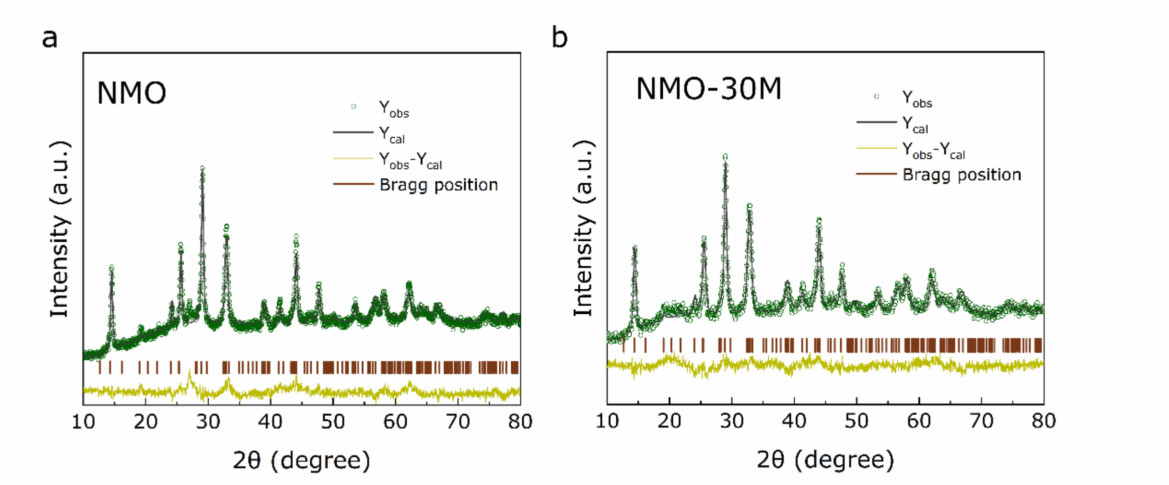

Fig. S2 Rietveld refinement plots for NMO and NMO-30M

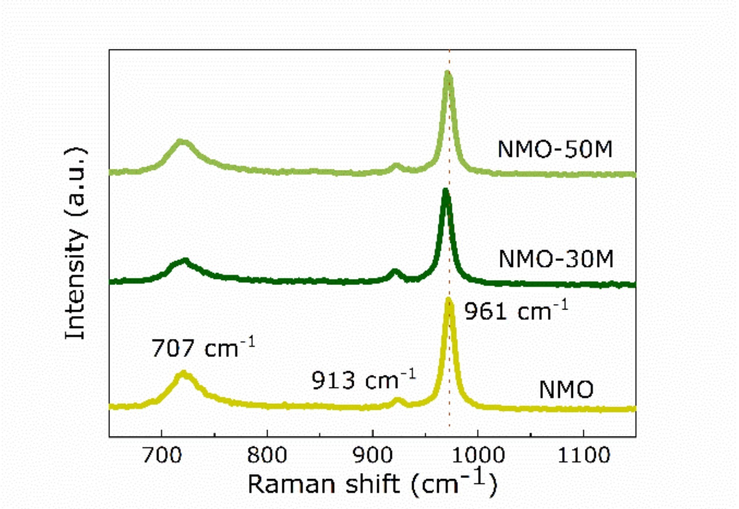

Fig. S3 Raman spectrum for NMO, NMO-30M and NMO-50M

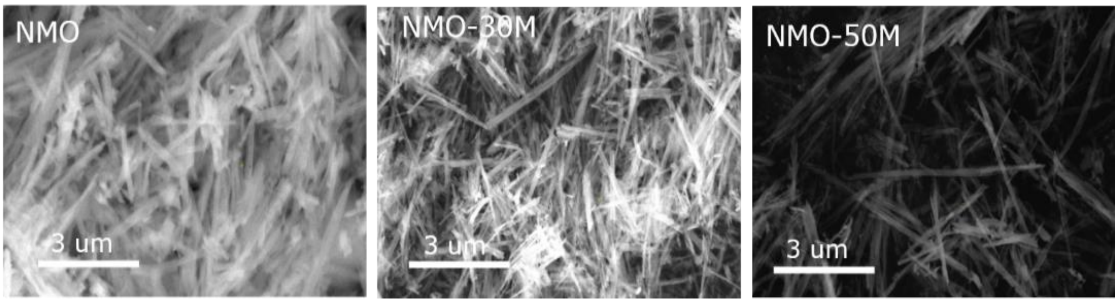

Fig. S4 Scanning electron microscope (SEM) images of NMO, NMO-30M and NMO-50M

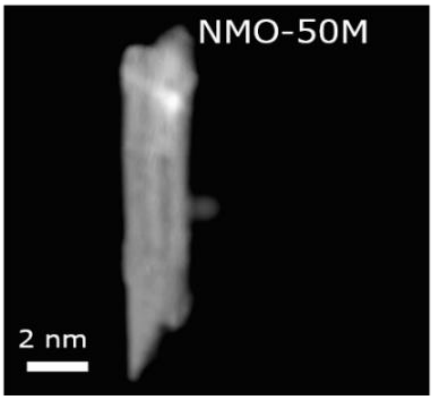

Fig. S5 TEM image of NMO-50M

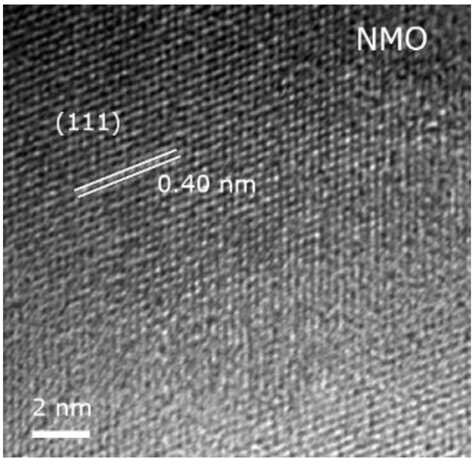

Fig. S6 HRTEM images of NMO

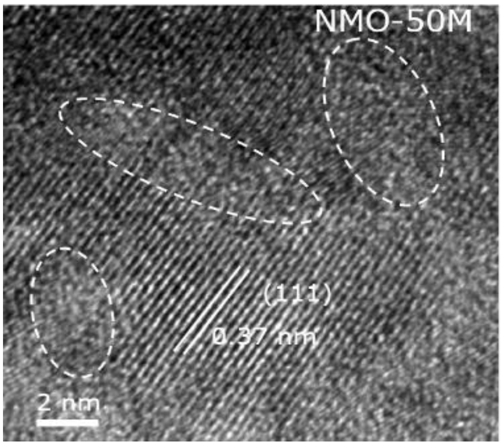

Fig. S7 HRTEM images of NMO-50M

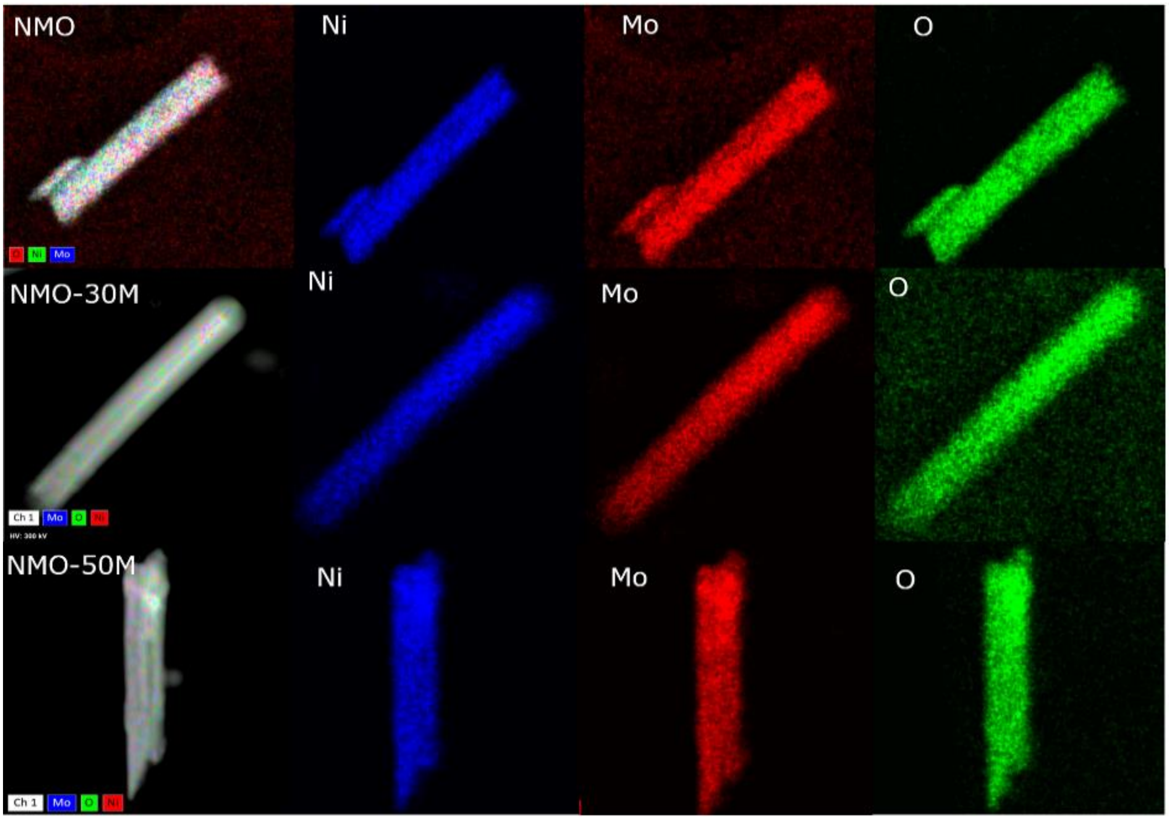

Fig. S8 EDX elemental mappings of NMO, NMO-30M and NMO-50M

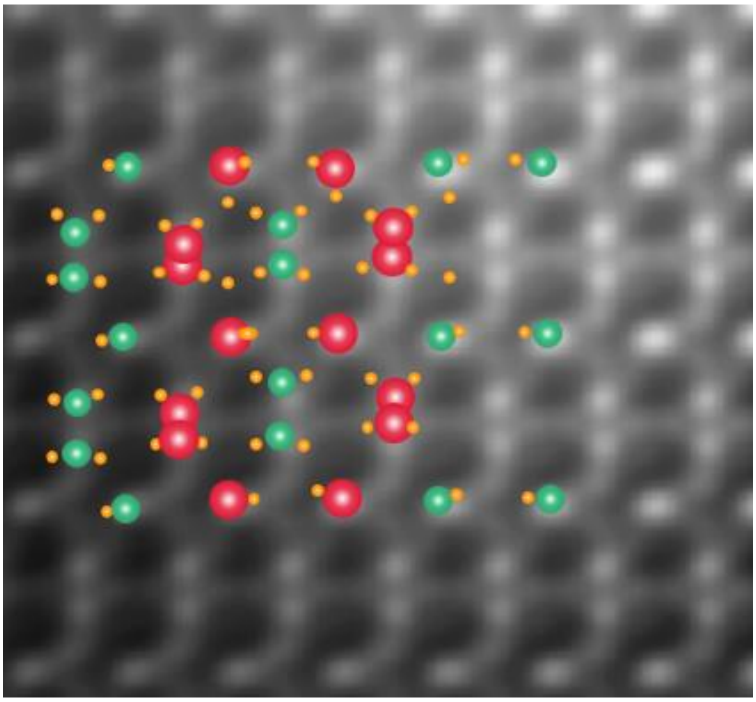

Fig. S9 HAADF-STEM images of NMO-30M

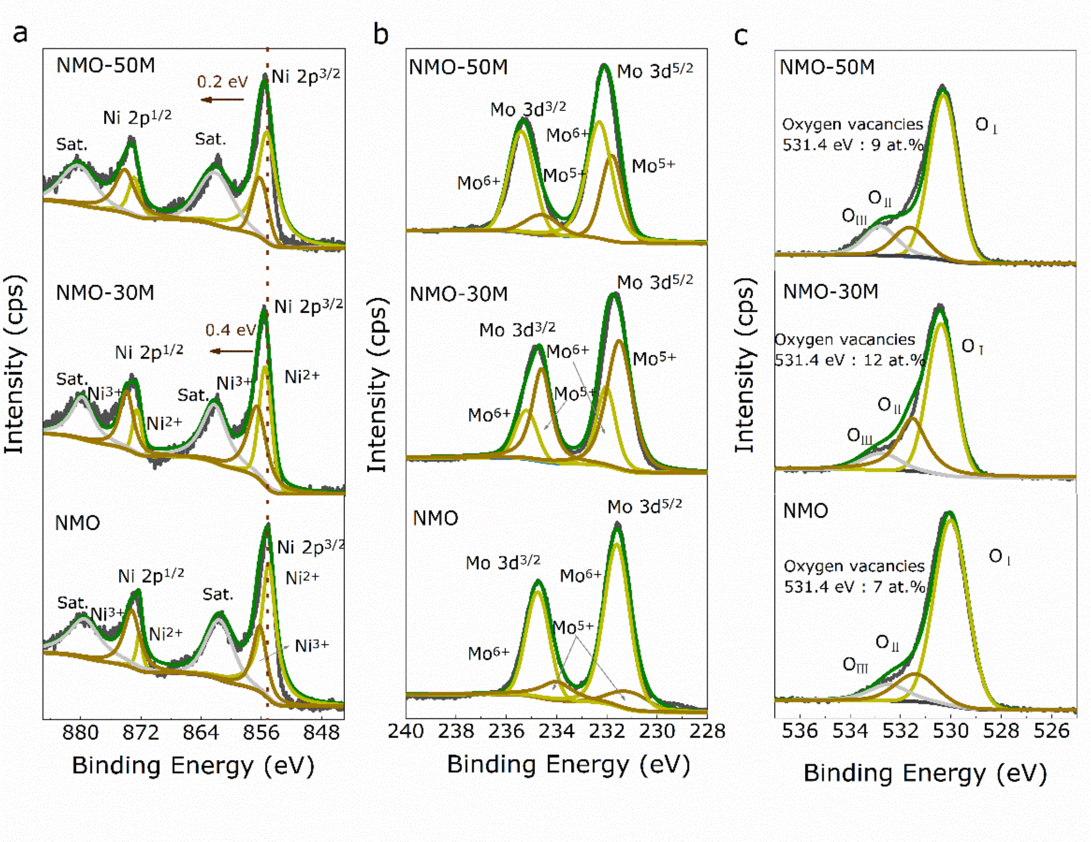

Fig. S10 High-resolution XPS spectra of (**a**) Ni 2p, (**b**) Mo 3d and (**e**) O 1s for NMO, NMO-30M and NMO-50M samples

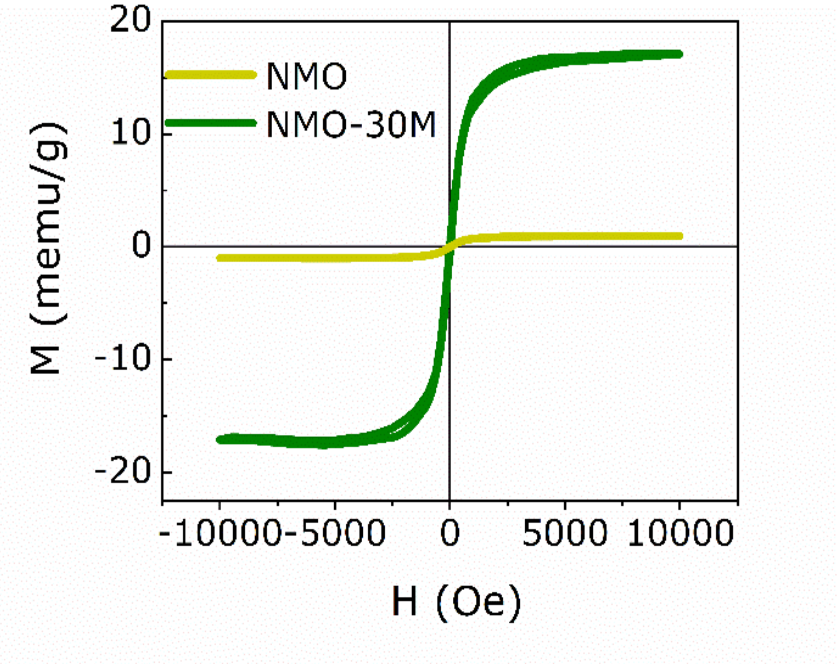

Fig. S11 Magnetic hysteresis loops of NMO and NMO-30M measured at room temperature

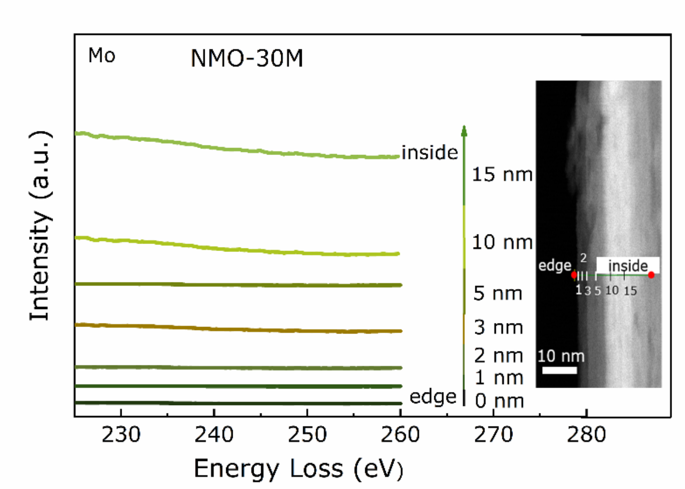

Fig. S12 Mo L-edge EELS spectra among different regions (STEM image) throughout etching

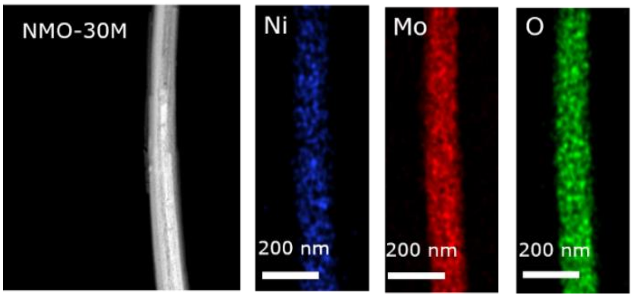

Fig. S13 HAADF image and elemental mappings of Ni, Mo and O

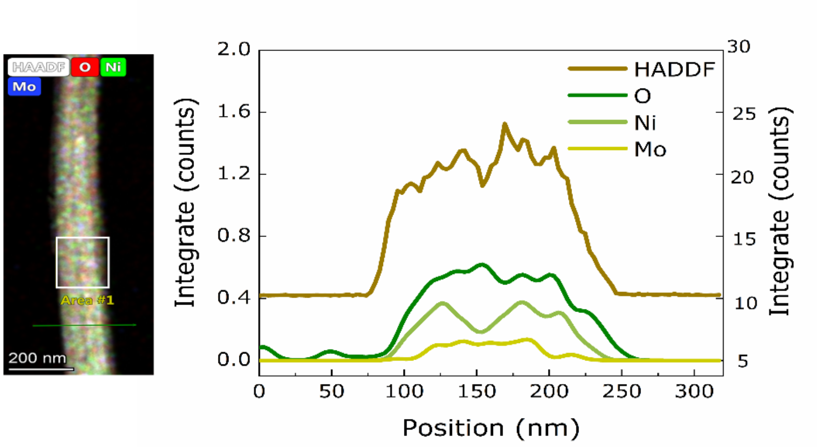

Fig. S14 The content of each element at the selected area

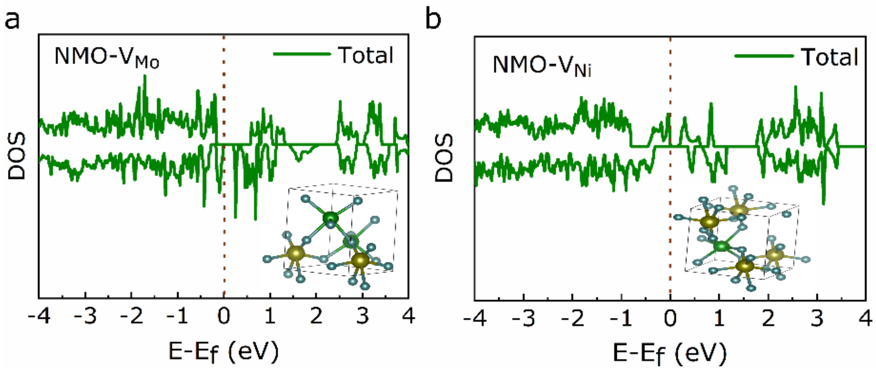

Fig. S15 DOS of (**a**) NMO-V_{Mo}, (**b**) NMO-V_{Ni} and the corresponding structure diagrams in the in inset

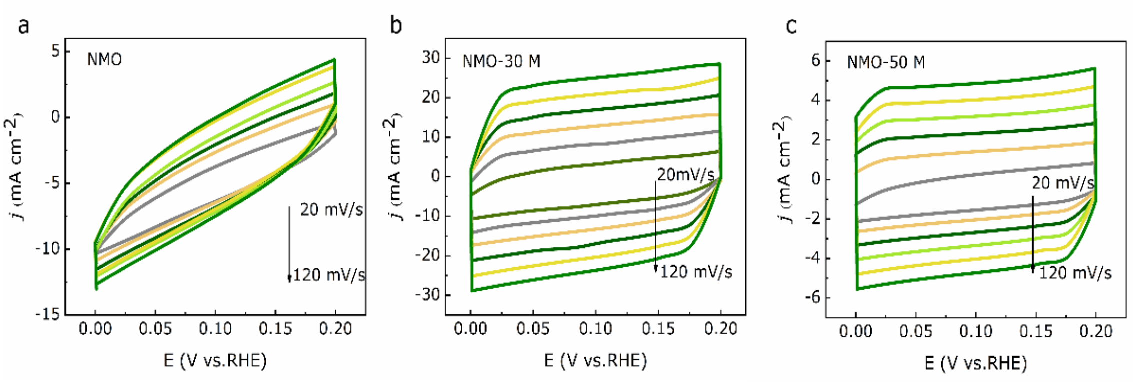

Fig. S16 Cyclic voltammetry curves at different scanning rates of NMO, NMO-30M and NMO-50M

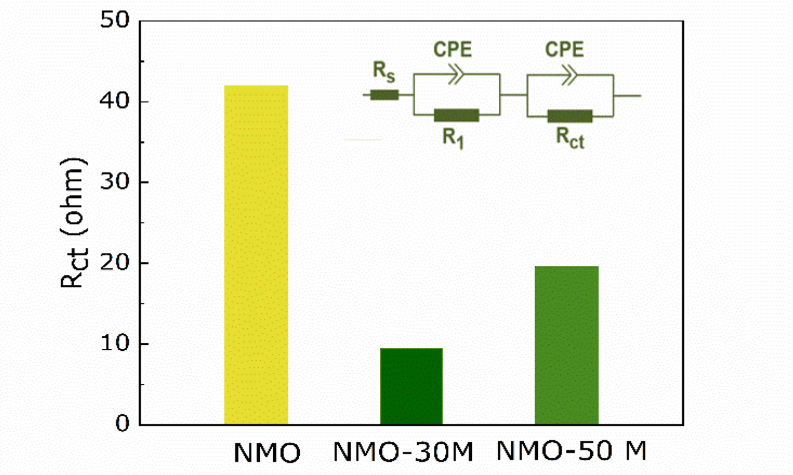

Fig. S17 The comparison of R_{ct} values for NMO, NMO-30M and NMO-50M

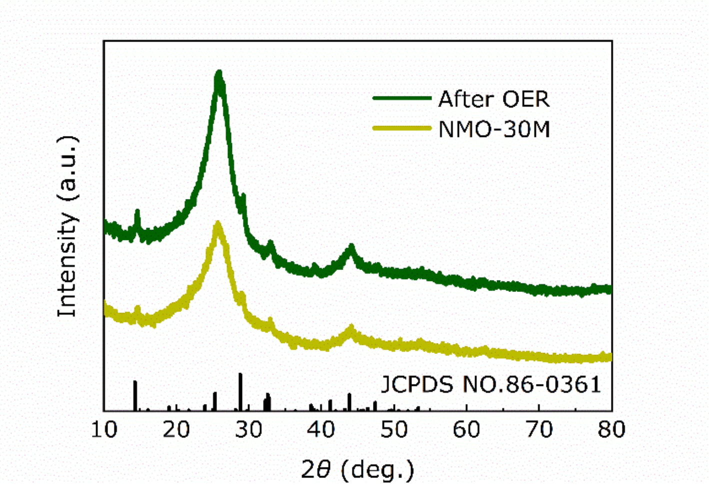

Fig. S18 XRD pattern of NMO-30M before and after OER

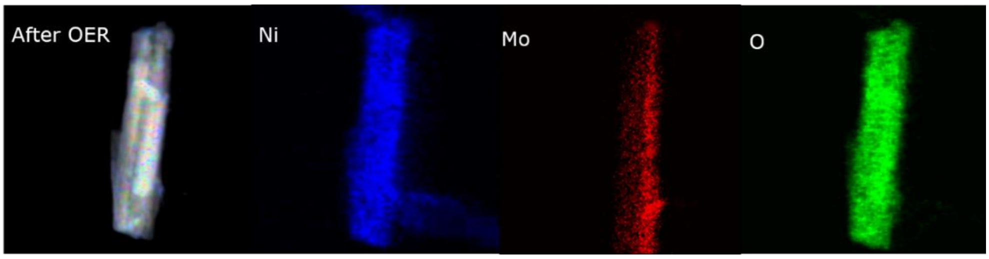

Fig. S19 DEX elemental mappings of NMO-30M after OER

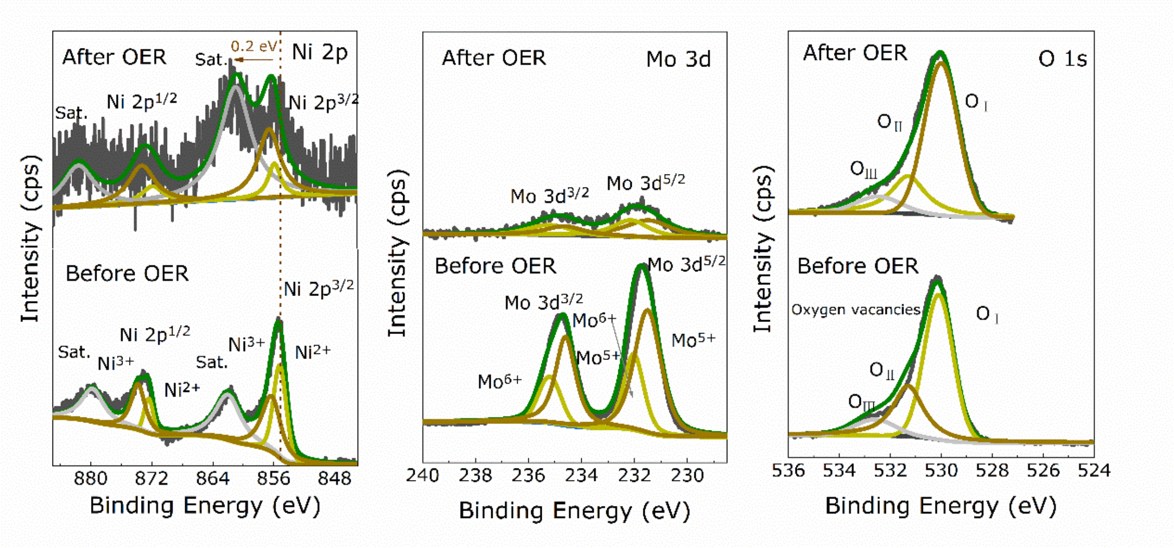

Fig. S20 XPS of NMO-30M before and after OER. (**a**) Ni 2p, (**b**) Mo 3d and (**c**) O 1s

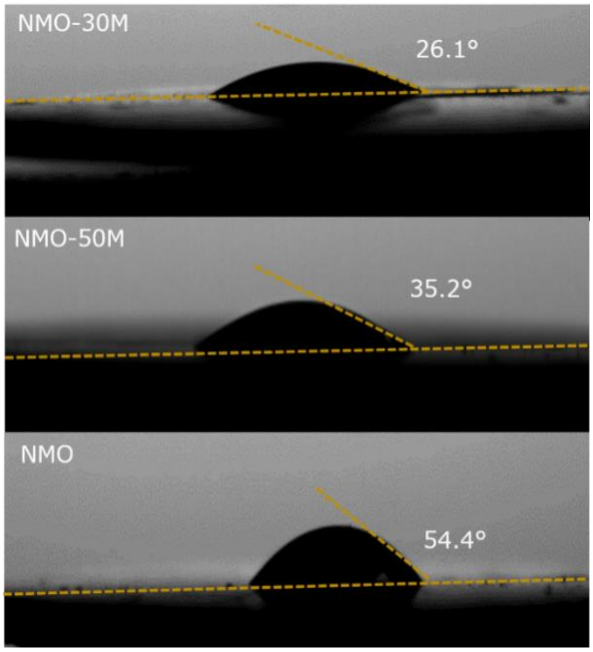

Fig. S21 Wettability measurements of NMO, NMO-30M and NMO-50M

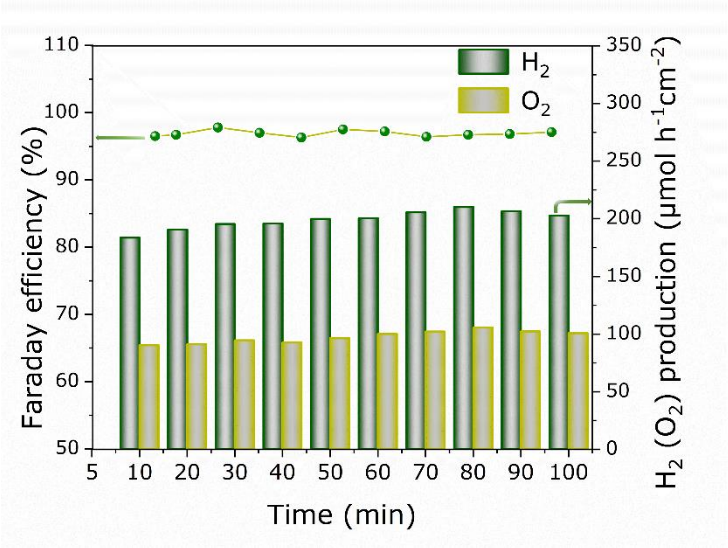

Fig. S22 The Faraday efficiency and H_2 (O_2) production rate of NMO-30M

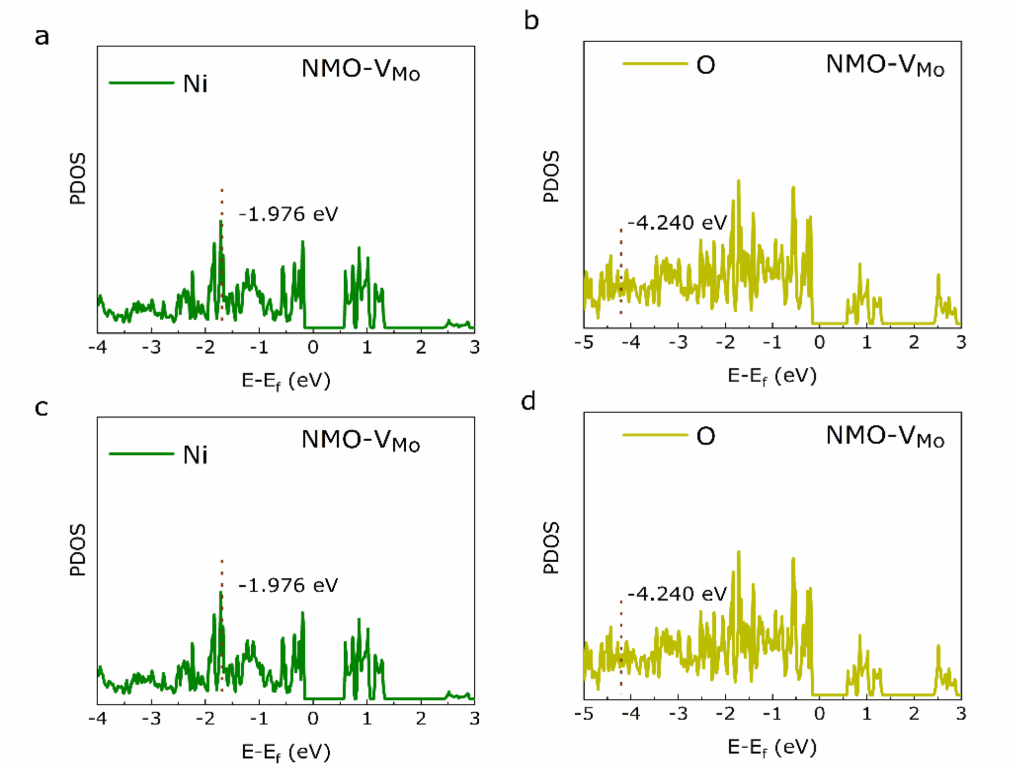

Fig. S23 The d-band center of NMO-V_{Ni} (**a-b**) and NMO-V_{Mo} (**c-d**)

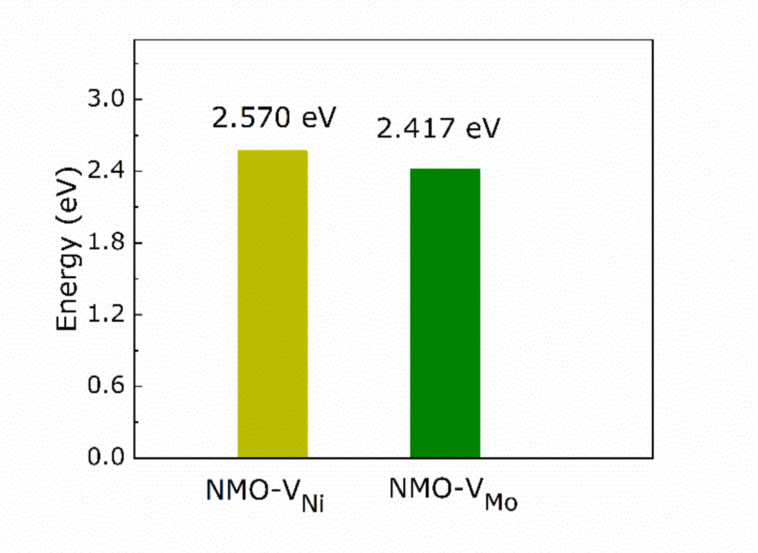

Fig. S24 The comparation of energy difference between the Ni d and O p band center for NMO-V_{Ni} and NMO-V_{Mo}

Table 1 ICP results of the $NiMO_4$ in different periods during etching process

Time (min)	The concentration of Ni vacancies (%)	The concentration of Mo vacancies (%)	The ratio of Ni:Mo
20	33.99	34.29	0.991
30	56.21	49.09	1.145
40	44.82	42.76	1.048
50	29.47	42.75	0.689

Table 2 The parameters of XRD Rietveld refinement for NMO and NMO-30M

	Lattice constant (Å)				Fitting parameters		
Sample	a	b	c	V (Å^3)	R_{wp}(%)	R_p(%)	χ^2
NMO	9.574	8.736	7.639	583.1	3.6	2.8	2.866
NMO-30M	9.590	8.760	7.652	586.4	3.1	2.5	2.265

Table S3 The surface compositions of NMO and NMO-30M derived from XPS test

Samples	O vacancies (O 1*s*)	Ni^{3+}/Ni^{2+} (Ni 2*p*)
NMO	7 at.%	0.43
NMO-30M	12 at.%	0.65
NMO-50M	9 at.%	0.52

Table S4 Bader charge of Ni cation in NMO and NMO-V_{NiMo}

Sample	NMO	NMO-V_{NiMo}
Ni	8.63 eV	8.40 eV

Supplementary References

[S1] J.K. Nørskov, J. Rossmeisl, A. Logadottir, L. Lindqvist, H. Jonsson et al. Origin of the overpotential for oxygen reduction at a fuel-cell cathode. J. Phys. Chem. B **108**(46), 17886-17892 (2004). https://doi.org/10.1021/jp047349j

[S2] J. Rossmeisl, Z.W. Qu, H. Zhu, G.J. Kroes, J.K. Nørskov, Electrolysis of water on oxide surfaces. J. Electroanal. Chem. **607**(1-2), 83-89 (2007). https://doi.org/10.1016/j.jelechem.2006.11.008

[S3] Á. Valdés, Z.W. Qu, G.J. Kroes, J. Rossmeisl, J.K. Nørskov, Oxidation and photo-oxidation of water on TiO_2 surface. J. Phys. Chem. C **112**(26), 9872-9879 (2008). https://doi.org/10.1021/jp711929d

[S4] J. Rossmeisl, A. Logadottir, J.K. Nørskov, Electrolysis of water on (oxidized) metal surfaces. Chem. Phys. **319**(1-3), 178-184 (2005). https://doi.org/10.1016/j.chemphys.2005.05.038

[S5] A.A. Peterson, F. Abild-Pedersen, F. Studt, J. Rossmeisl, J.K. Nørskov, How copper catalyzes the electroreduction of carbon dioxide into hydrocarbon fuels. Energy Environ. **3**(9), 1311-1315 (2010). https://doi.org/10.1039/c0ee00071j
